# Observation of the Peach Fruit Moth, *Carposina sasakii*, Larvae in Young Apple Fruit by Dedicated Micro-Magnetic Resonance Imaging

**DOI:** 10.1673/031.010.14105

**Published:** 2010-09-10

**Authors:** Mika Koizumi, Fumio Ihara, Katsuhiko Yaginuma, Hiromi Kano, Tomoyuki Haishi

**Affiliations:** ^1^ Research Institute for Science and Engineering, Waseda University, 2-2 Wakamatsu-cho, Shinjyuku, Tokyo 1628480, Japan; ^2^ National Institute of Fruit Tree Science, National Agriculture and Food Research Organization, 2-1 Fujimoto, Tsukuba, Ibaraki 305-8605, Japan; ^3^ Oak-Hill Georgie Patch-Work Laboratory, 4-13-10 Miyamoto, Funabashi, Chiba 273-0003, Japan; ^4^ MR Technology, Inc., 2-1-6 Sengen, Tsukuba, Ibaraki 305-0047, Japan

**Keywords:** growth, infestation, movement, small MRI apparatus with a permanent magnet

## Abstract

Infestation of young apple fruits by the larvae of the peach fruit moth, *Carposina sasakii* Matsumura (Lepidoptera: Carposinidae), was studied by a small dedicated micro-magnetic resonance imaging (MRI) apparatus using the three-dimensional (3D) gradient-echo method and the two-dimensional (2D) and 3D spin-echo methods. Changes from a young larva at 1.8 mm in length to a mature one ready to leave the fruit were observed in relation to the progression of infestation of the fruit tissues. The trace of larva intrusion was demonstrated by a series of sliced images in the 3D image data of an infested fruit, where it entered from outside the calyx, and migrated to near the vasculature around the carpel through the core. The small, dedicated MRI device was proven useful for ecological studies of the growth and movement of insect larvae in their food fruits. It can also be applied to detect the infestation of small fruits by insect larvae.

## Introduction

The peach fruit moth, *Carposina sasakii* Matsumura (Lepidoptera: Carposinidae), is a harmful, primary insect of apple fruits, the infestation of which is prevented by spraying insecticides and air permeation of hormones for mating disruption. The fruits are also carefully checked before they are sent to markets. These processes are elaborately programmed throughout the growing season of the fruits in order to preserve the soundness and safety of the products, based on the ecological observations of the moth (Narita 1986). However, the behaviour of the larva in its food fruit has not yet been clarified.

Damages or defects in apple fruits (e.g., watercore, mealiness, and bruising) are nondestructively detected by magnetic resonance imaging (MRI) (Wang et al. 1988; McCarthy et al. 1995; Clark et al. 1998; Barreiro et al. 1999). MRI is considered a potential means for studying the growth, movement, and hostparasite interaction of insects (Hart et al. 2003). It has been used to study the developmental changes of lepidopteran pupae (*Pieris brassicae* and *Graphiphora augur*) (Goodman et al. 1995), the morphology of the diving beetle (*Dytiscus marginales*) (Wecker et al. 2002), common wasp (*Vespula vulgaris*) queen and large ant (*Dinoponera quadriceps*) worker (Hart et al. 2003), the host-parasitoid interaction of the seven-spot ladybird (*Coccinella 7-punctata*) infected with small parasitic wasps (*Dinocampus coccinellae*) (Chudek et al. 1998), the development of the desert locust (*Schistocerca gregaria*) embryo (Gassner and Lohman 1987), and dynamics of circulatory, respiratory, and digestive systems in pupae of the tobacco hornworm (*Manduca sexta*) (Hallock 2008). Therefore, it was proposed that the growth of the larvae of the peach fruit moth associated with the expanding infestation in apple fruits could be observed by MRI.

In the current investigation, the firstgeneration larvae artificially reared and infested young apple fruits just after fruit setting were examined during the growth stages, and the path of a larva intruding from the outside to a place near the vasculature around the carpel of a fruit was observed with the use of a small dedicated MRI device, developed for the research of food science and agriculture (Koizumi et al. 2006, 2008). This apparatus provided a unique means of detecting infestation by insect larvae of small fruits, as well as studying ecological aspects of insect larvae in their food fruits.

## Materials and Methods

### Infested young apple fruits

Young apple, *Malus domestica* Borkhausen (Rosales: Rosaceae) fruits were collected during the last 10 days of May in 2007, after fruit setting in the non-pesticide field of the National Institute of Fruit Tree Science, Apple Research Station (Morioka, Japan). Young fruits that were smaller than 30 mm in diameter and on which *C. sasakii* laid eggs were selected and incubated for hatching in a room at 20° C under 16:8 L:D for 2 weeks. The fruits infested by larvae were then examined by MRI 10 days, 15 days, and 25 days after hatching. Measurements were made from 25 June 2007 to 10 July 2007.

### MRI measurements

A small dedicated MRI apparatus with a 1 Tesla (T) permanent magnet (MR Technology, Inc.) was used (Koizumi et al. 2006). The resonance frequency was 42.58 MHz (^1^H); the field of view was 30 mm in diameter, and the magnitudes of gradient magnetic field were Gx = 40 mT/m, Gy = 35 mT/m, and Gz = 52 mT/m.

**Figure 1.**
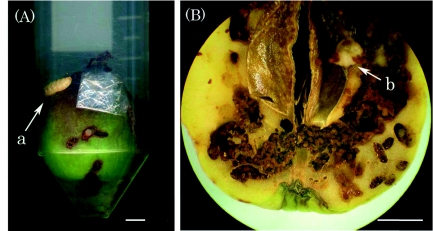
(A) Young apple fruit in a sample holder. (B) Larva and infested holes in the fruit tissues with accumulated excreta. A young apple fruit was placed in the sample holder of a plastic test tube 30 mm in diameter. The infested apple fruit was observed by a low-magnification optical microscope after the completion of MRI measurements, (a) A larva going out of the fruit and (b) a larva with accumulated excreta. The scale bars are 5 mm. High quality figures and videos are available online.

An infested fruit was placed in a 30 mmdiameter plastic test tube, fastened with pieces of vinyl sheet (Figure 1A), and inserted into the measurement cell of the MRI apparatus. Measurements were made by the threedimensional (3D) gradient-echo method, and the two-dimensional (2D) and 3D spin-echo methods (Callaghan 1991). Measurement conditions for detecting larvae and infested holes in apple tissues were optimized by changing flip angles of the radio frequency for the 3D gradient-echo method and by changing repetition time (TR) and echo time (TE) for the 2D spin-echo method. TR of 0.1 s, TE of 2.18 ms, and flip angle of 30° were found to be suitable for the 3D gradient-echo method; and TR of 0.8 s and TE of 7 ms were found to be suitable for the 3D spin-echo method. Three fruits were measured by the 3D gradient-echo method according to the progression of infestation, and a fruit was examined by the 3D spin-echo method to trace larval intrusion into the fruit. Four transient acquisitions were accumulated and images were created on a matrix of 128 × 128 × 128 with a spatial resolution of 234 µm or a matrix of 256 × 256 × 256 with a resolution of 117 µm. Images were further processed by the ImageJ program (a public domain Java image processing program (version 1.33)), available on the Internet at http://rbs.info.nih.gov/ij/.

### Observation by an optical microscope

Insects, infested holes, and accumulated excreta were confirmed using a lowmagnification optical microscope after the completion of measurement (Figure 1B).

## Results

### Optimization of measurement conditions

**Figure 2.**
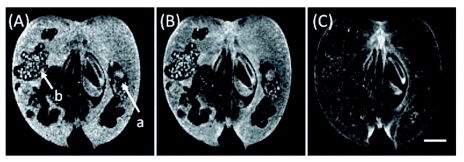
Image changes of a heavily infested apple fruit measured by the 3D gradient-echo method using 15° (A), 30° (B), and 90° (C) flip angles. The scale bar is 5 mm. Arrow a indicates larva and arrow b indicates excreta on the image (A). TR was 0.1 s and TE was 2.18 ms. The resolution was 234 µm. High quality figures and videos are available online.

**Figure 3.**
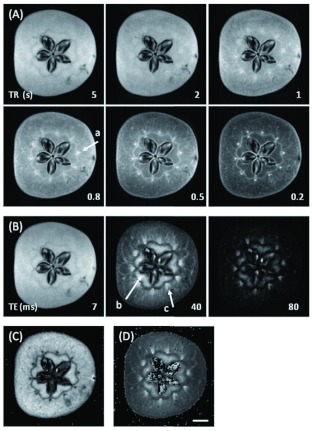
Series of *T*_1_-weighted images (A) measured using TE of 7 ms and changing TR from 5 s to 0.2 s. *T*_2_-weighted images (B) using TR of 5 s and changing TE from 7 ms to 80 ms. *T*_1_ value image (C) constructed from the data of *T*_1_-weighted images (A), and *T*_2_ value image (D) constructed from the data of *T*_2_-weighted images (B). The scale bar is 5 mm. Arrow a indicates a vascular bundle; arrow b core tissue, and arrow c outside vascular bundle. High quality figures and videos are available online.

The measurement conditions for detecting larvae and infested holes in apple tissues were examined by changing TR, TE, and flip angle (the flip angle was tested in only the gradientecho method). Figure 2 presents images of a heavily infested fruit measured by the 3D gradient-echo method using flip angles of 15° (A), 30° (B), and 90° (C). TR was 0.1 s and TE was set at the shortest period (2.18 ms) available in the gradient-echo method for the apparatus because the spin-spin relaxation times (*T*_2_) of sarcocarp tissues were short (Figure 3 D). The image acquired with a 15° flip angle clearly contrasted between infested holes, a larva (arrow a), excreta (arrow b), and the fruit tissues. However, the background of the image was noisy because of the low signal intensity. Infested holes, excreta, and a larva were not clearly distinguished from the fruit tissues in the image taken with a 90° flip angle. Images acquired by 30° or 45° flip angles were suitable for studying infestation; the former provided stronger echo signals than the latter.

Images of an apple fruit in an early stage of infestation (10 days after hatching) were obtained by the 2D spin-echo method with TE of 7 ms, the shortest echo time useable for the apparatus, and by shortening TR from 5 s to 0.2 s, that is, a series of spin-lattice relaxation time (*T*_1_)-weighted images (Figure 3A). Vascular bundles (arrow a) were emphasized when TR was decreased to less than 1 s, but the infested holes could not be clearly differentiated from sarcocarp tissue. In a series of *T*_2_-weighted images measured with TR of 5 s and by elongating TE from 7 ms to 80 ms, sarcocarp tissue signals decreased significantly at 40 ms, so that core tissues (arrow b) together with outside vasculature (arrow c) of core were clarified (Figure 3B), but not infested tissues. The *T*_1_ value image (0 to 1 s, Figure 3C) was constructed using the data in Figure 3 A, and the *T*_2_ value image (0 to 80 ms, Figure 3D) was constructed using Figure 3B. *T*_1_ values were high in sarcocarp and core tissues, and low in the border of the core, seeds, and vascular bundles. In contrast, *T*_2_values were low except for core tissues, some seeds, and outside vascular bundles. Larvae were not obvious in the images acquired by the 2D spin-echo method because slice thickness (3.6 mm) was too great to clearly detect small larva of early infestation. Signal intensities of larvae were similar to those of sarcocarp tissue (Figure 5).

### Observation of larval growth by the 3D gradient-echo method

**Figure 4.**
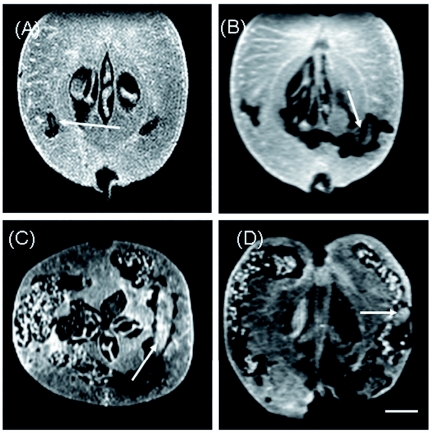
Images of *Carposina sasakii* larvae at various growth stages measured by the 3D gradient-echo method. TR was 0.1 s; TE 2.18 ms, and 30° flip angle. Images were properly rotated in order to depict the larvae by using the ImageJ program. The scale bar is 5 mm. (A) 1.8 mm-long larva. (B) Growing 5-mm-long larva. (C) Grown larva exceeding 10 mm in length. (D) Head of a mature larva just exiting the fruit. The larvae are indicated by arrows. The resolution was 117 µm (A, C) or 234 µm (B, D). High quality figures and videos are available online.

Figure 4 presents the images of larvae at various growth stages in young apple fruits at 10 days (A and B), 15 days (C), and 25 days (D) after hatching. The images were measured by the 3D gradient-echo method and were properly rotated in order to depict the larvae in the infested tissues, with the use of theImageJ program. Infested holes and a small (1.8 mm-long) larva were observed in the sarcocarp of a fruit 10 days after hatching (Figure 4A). A growing 5 mm-long larva was found in the lower right sarcocarp of another fruit 10 days after hatching (Figure 4B), or where infestation had proceeded, signals in holes almost completely disappeared, and small amounts of excreta were detected in the holes. Infestation of tissues advanced, and a larva more than 10 mm in length was detected 15 days after hatching (Figure 4C). Large amounts of sawdust-shaped excreta were accumulated in the infested holes, and the full-length figure of the larva could not be depicted in a sliced image. The head of a larva of the last growth stage that was going to leave the fruit was observed at 25 days after hatching (Figure 4D). Most of the fruit tissues were caved by the larva, and concentrated excreta were found. Larvae could be clearly detected in the dark infested holes when they were in the early growth stages. However, large larvae were not distinguished from excreta in the later growth stages unless their figures were distinctive; therefore, images had to be rotated to find the larvae.

### Trace of larval intrusion into an apple fruit by the 3D spin-echo method

**Figure 5.**
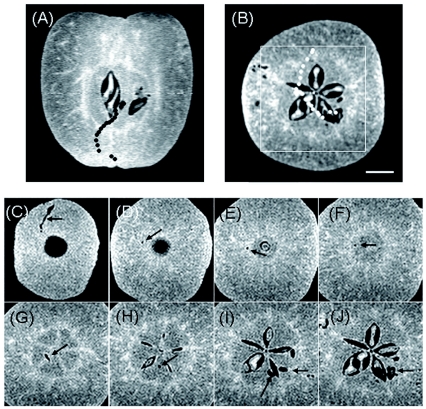
Intrusion marks of a small larva from the outside to the final place in an apple fruit. (A) vertical and (B) horizontal images of the infested fruit. The trace of intrusion is plotted with (A) black circles on the images and (B) white circles on the image by using the slice images at every 5^th^ interval in the original 3D image data measured by the spin-echo method. TR was 0.8 s, and TE was 7 ms. The areas selected on the horizontal image (B) by a white square are presented in the images from C to J at every 10^th^ interval. Small infested holes are indicated by arrows. The scale bar is 5 mm. High quality figures and videos are available online.

A young apple fruit 10 days after hatching was measured by the 3D spin-echo method (Figure 5). A small (2.5 mm long) larva was observed in the hole of the right core of the fruit in a vertically sliced image (Figure 5A), and the same larva was observed in the hole adjacent to the seed at the lower right of the core in a horizontal image (Figure 5B). A continuous cave was found from the 36^th^ section to the 106^th^ section upward, corresponding to the bottom half of the fruit, in a series of 256 sections of 3D image data. The marks indicated larva intrusion into the fruit. The central areas denoted by a white square in Figure 5B were presented from the sectional images at 10^th^ intervals (Figures 5C, 5D, 5E, 5F, 5G, 5H, 5I, 5J). The larva entered the fruit from outside the calyx and migrated to the calyx side (Figure 5C), climbed just above to the core (Figures 5D, 5E), traversed the core in an ascending path under the seeds (Figures 5F, 5G, 5H), and then intruded into the infesting hole through the carpel (Figures 5I, 5J). The pathway is plotted with black circles in Figure 5A and white circles in Figure 5B, using the sectional images at 5^th^ intervals in the original data of the 3D measurement. The larva travelled through a cave smaller than 400 µm in diameter from the entrance to near the vasculature around the carpel of the fruit through the central areas of the core. The pathway of the larva intrusion can be visualised in the Video 1, scanning the horizontally sliced images in a series of 256 sections of the original 3D image data of Figure 5 from the 36^th^ section (bottom of the fruit) to the 106^th^ section (near the center of the fruit), although time scale of the video does not represent true time-lapse.

## Discussion

It was possible to use MRI for non-destructive detection of the larvae of *C. sasakii* in their food apple fruits (Ihara et al. 2008). The enlargement of injured tissues in the fruit was clearly depicted, as was expected from research on watercore, mealiness, and bruising (Wang et al. 1988; McCarthy et al. 1995; Clark et al. 1998; Barreiro et al. 1999). Using the gradient-echo method, the growth of the larvae was observed in relationship to the increase of infestation in young apple fruits. Intense signals were obtained using a short TR (0.1 s) because of a short TE (2.18 ms) by the 3D gradient-echo method. The image contrast was high but the morphology contour was not so sharp (Figures 2, 4) as those acquired by the 3D spin-echo method (Figure 5). A flip angle of 30° provided the proper contrast in images required for detecting infested holes, larvae, and excreta.

A tiny (approximately 2 mm long) larva was detected (Figure 4A), and its growth was associated with the increase of infested areas in the sarcocarp tissues (Figure 4B). Grown larva carved large holes in the sarcocarp tissues, where large amounts of excreta accumulated (Figure 4C). The larvae exited the fruit after they became mature (Figure 4D). The spatial resolutions of images were appropriate to locate the larvae (Gassner and Lohman 1987) and to distinguish individual tissues of the young apple fruits, although it was impossible to observe the morphological details of the insect (Goodman et al. 1995; Chudek et al. 1998; Wecker et al. 2002). It took 2 h to measure a set of 3D image data by the gradient-echo method. The measurement time was not so short to trace dynamics of insect metabolism (Hallock 2008), but adequately short to observe the growth and movement of the larvae. The gradient-echo method of the small dedicated MRI apparatus was therefore useful in ecological studies of the dynamic changes of insects in infested fruits (Hart et al. 2003).

Tissues in fruits as well as small infested holes were amply depicted by the 2D spinecho method in spite of the thick slices because the images were rich in gradation and distinctive in the contour of morphology (Figure 3A). Long TRs exceeding 1 s were preferable in the measurements because of the long *T*_1_ of sarcocarp tissue (Figure 3C), while the shortest TE (7 ms) was necessary due to the steep decline of signals (Figure 3B) or short *T*_2_ in sarcocarp tissue (Figure 3D). Accordingly, long times were necessary in the 3D spin-echo measurements. Based on the results in Figure 3, the shortest TR (0.8 s) that enabled obtaining the required contrast for detecting small infested holes within a practical time was employed as a measurement condition (Figure 5). Although measurement took as long as 15 h, the images acquired by the 3D spin-echo method were sharp and clear, and thus suitable for detailed examination of the marks of larva movement.

An intruding pathway of a very small larva was visualised and a larva was detected in a hole (Figures 5A, 5B). The infesting larva travelled the long distance from the entrance to the core, not staying in one place, through caves less than 400 µm in diameter after intrusion into the fruit (Figures 5C, 5D, 5E, 5F, 5G, 5H, 5I, 5J; Video 1). It then stopped near the vasculature around the carpel, where a watercore developed after the fruit ripened on the mother tree (Marlow and Loescher 1984; Clark et al. 1998), and sugars were probably first delivered. These findings indicated that the resolution of the 1 T small dedicated MRI apparatus was adequate for detecting the first instar larvae just after they enter the fruit (Chudek et al. 1998). The movement of the larvae travelling through apple tissues can be non-destructively observed by the apparatus without affecting the organic architectures of the narrow and fragile tunnels and without the complicated preparation of thin sections of materials required for optical observations. In addition, MRI allows repeated measurement of the same infested fruits for investigating the advance of infestation (Hart et al. 2003; Ihara et al. 2008).

The 1 T dedicated MRI device is small, easy to operate and maintenance-free; it can be used for a specified subject without restriction of operation times by placing it in a lightly air-conditioned ordinary research room (Koizumi et al. 2006, 2008). Since the measurement cell was just 30 mm in diameter, the apparatus could not deal with harvested apple fruits, which is the primary objective for preventing moth infestation. The infested holes in harvested apples were detected by a 0.2 T dedicated MRI, which had a wider pole gap (160 mm) and was equipped with a larger detection coil (110 mm) (Haishi et al. 2009). In contrast, it was not possible to find the small larvae of the early growth stages in the harvested apple fruits using the 0.2 T apparatus, due to rough resolution from the principles of MRI. In this context, the combination of the 1 T small dedicated MRI device and the infested young apple fruits is considered to be a potential system for exploring new standpoints in the ecological study of the growth and movement of the larvae of *C. sasakii* in their food fruits. The apparatus can also be used to detect the infestation of other small fruits by noxious insect larvae.

**Video 1.**
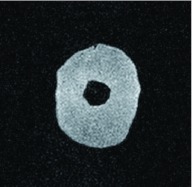
A video showing the trace of *Carposina sasakii* larva intruding into a young apple fruit which corresponds to Figure 5. Horizontally sliced images in a series of 256 sections of 3D image data are scanned from 36^th^ section (bottom of the fruit) to 106^th^ section (near center of the fruit), and the continuous marks of larva infestation are found. The infesting larva travelled the long distance from the entrance to the core, not staying in one place, through caves less than 400 µm in diameter after intrusion into the fruit. It then stopped near the vasculature around the carpel. High quality figures and videos are available online.

## Abbreviations

MRI: magnetic resonance imaging;
TR: repetition time;
TE: echo time;
2D: two-dimensional;
3D: three-dimensional
